# Canavanine Increases the Content of Phenolic Compounds in Tomato (*Solanum lycopersicum* L.) Roots

**DOI:** 10.3390/plants9111595

**Published:** 2020-11-17

**Authors:** Pawel Staszek, Urszula Krasuska, Magdalena Bederska-Błaszczyk, Agnieszka Gniazdowska

**Affiliations:** 1 Department of Plant Physiology, Institute of Biology, Warsaw University of Life Sciences-SGGW, Nowoursynowska 159, 02-776 Warsaw, Poland; urszula_krasuska@sggw.edu.pl; 2 Department of Botany, Institute of Biology, Warsaw University of Life Sciences-SGGW, Nowoursynowska 159, 02-776 Warsaw, Poland

**Keywords:** canavanine, phenolic compounds, oxidative stress, nitric oxide, PAL, phenolic oxidase

## Abstract

Canavanine (CAN) is a nonproteinogenic amino acid, and its toxicity comes from its utilization instead of arginine in many cellular processes. As presented in previous experiments, supplementation of tomato (*Solanum lycopersicum* L.) with CAN led to decreased nitric oxide (NO) level and induced secondary oxidative stress. CAN improved total antioxidant capacity in roots, with parallel inhibition of enzymatic antioxidants. The aim of this work was to determine how CAN-dependent limitation of NO emission and reactive oxygen species overproduction impact content, localization, and metabolism of phenolic compounds (PCs) in tomato roots. Tomato seedlings were fed with CAN (10 and 50 µM) for 24 or 72 h. Inhibition of root growth due to CAN supplementation correlated with increased concentration of total PCs; CAN (50 µM) led to the homogeneous accumulation of PCs all over the roots. CAN increased also flavonoids content in root tips. The activity of polyphenol oxidases and phenylalanine ammonia-lyase increased only after prolonged treatment with 50 µM CAN, while expressions of genes encoding these enzymes were modified variously, irrespectively of CAN dosage and duration of the culture. PCs act as the important elements of the cellular antioxidant system under oxidative stress induced by CAN.

## 1. Introduction

Reactive oxygen species (ROS), as products of incomplete reduction of oxygen, are formed in every cell of living organisms. The toxic effects of ROS on the structural elements of the cell (proteins, lipids, nucleic acids) have been known for a long time. However, in the last decades, more and more has been understood about their signaling role, pointing at an important function in modeling the plant response to environmental conditions. The induction of oxidative stress due to the overproduction of ROS is considered as a common response of plants to various stress factors [1].

The cellular antioxidant system could counteract oxidative stress by scavenging ROS. The main components of the enzymatic antioxidant system are superoxide dismutase (SOD), catalase (CAT), and peroxidase (POx). Among the enzymes controlling the concentration of ROS, the enzymes of the Halliwell-Asada cycle (glutathione reductase (GR), monodehydroascorbate reductase, dehydroascorbate reductase, ascorbate peroxidase), which determine the appropriate redox state of low-molecular antioxidants, are also important. For proper action of the antioxidant system, low-molecular nonenzymatic antioxidants such as glutathione, ascorbate, tocopherols, and carotenoids are necessary [2].

Phenolic compounds (PCs) are endogenous growth regulators which can modulate physiological processes such as membrane permeability, vesicle trafficking, gene transcription, and signal transduction in plants. PCs, according to the rule proposed by Quideau et al. [3], are natural secondary metabolites, originating from the shikimate/phenylpropanoid or malonate pathways. PCs refer to a wide group of chemicals such as phenolic acids, flavonoids, phenocarbonic acids, stilbenes, lignans, and polymeric lignans [4]. Phenylalanine ammonia-lyase (PAL, EC 4.3.1.15) is a key enzyme in PCs biosynthesis [5]. It catalyzes the nonoxidative elimination of the -NH_2_ group from L-phenylalanine (Phe) to form *trans*-cinnamate, which has one aromatic ring and is considered as the starting molecule for the synthesis of other PCs [6].

Flavonoids and their conjugates are the largest group of PCs. Robards and Antolovich [7] estimated that about 2% of all assimilated carbon is converted into flavonoids and closely related molecules. Flavonoids were found in different plant tissues, both inside the cells as well as on the surface of plant organs [8].

PCs are known to enhance or inhibit oxidative stress [5]. One of the most significant PCs properties is their capacity to chelate metal ions. PCs can chelate copper and iron ions due to the existence of suitable functional groups: carboxyl and hydroxyl. Chelation of metal ions by PCs can diminish hydroxyl radical formation due to the Fenton reaction. Flavonoids are found to be the most efficient class of chelating agents among PCs [9]. Furthermore, PCs (especially flavonoids) can stabilize biological membranes by reducing their fluidity, which limits the diffusion of free radicals and as a result decreases peroxidation of membrane lipids [10]. Membrane stabilization results from the ability of PCs (particularly flavonoids) to bind to some integral membrane proteins and phospholipids [11]. PCs have also the potential to interact with proteins. This property allows, among others, the inhibition of the enzymes involved in radicals generation (e.g., xanthine oxidase, lipoxygenases, cytochrome P450) [5].

Polyphenol oxidases (PPOs) (EC 1.10.3.2) are a group of copper-containing enzymes which catalyze the O_2_-dependent oxidation of *ortho(o)*-phenolics to *o*-quinones [12]. Then, the secondary reaction of quinones (polymerization and crosslinking) can lead to the formation of polymeric dark pigments. The role of PPOs in the process of food (fruits and vegetables) browning is very well characterized [13]. However, knowledge about PPOs’ role in plant development and responses to stresses is far less documented. PPOs are expressed differently in shoots, stems, leaves, and roots according to the expression of multigene PPOs family [14,15]. The best characterized PPOs are induced in the defense reaction against herbivorous insects. PPOs activity leads to the formation of very reactive quinones, resulting in the lowering of the nutritive value of proteins [16]. There are also some studies on the antioxidant activity of chloroplastic PPO during photosynthesis but the results are complex and inconclusive [14].

Production of secondary metabolites (including PCs) is stimulated by nitric oxide (NO) [17]. NO is an important gaseous signaling molecule that regulates many developmental processes in plants and is involved in plant reaction to stresses [18]. Enhanced emission of NO in response to biotic and abiotic stresses plays an important role in the induction of the synthesis of secondary metabolites. Durner et al. [19] demonstrated that NO increased transcription of the genes encoding PAL in tobacco (*Nicotiana tabacum* L.).

Canavanine (CAN, L-2-amino-4-guanidooxy-butanoic acid) is one of over 1000 nonproteinogenic amino acids (NPAAs) which are synthesized in plants. CAN production is restricted to some Fabaceae species. Seeds of genera *Canavalia*, *Dioclea*, and *Hedysarum*, as well as seeds and sprouts of alfalfa (*Medicago sativa* L.), are known as a reach source of CAN. This NPAA acts as an essential nitrogen storage compound but also plays a protective role, mostly against seed predators [20]. CAN is a structural guanidinoxy analogue of L-arginine (Arg) and is considered as an Arg antimetabolite. In enzymatic reactions utilizing Arg as a substrate, this proteinogenic amino acid can be replaced by CAN. Arginyl tRNA synthetase easily esterifies CAN to the tRNA^Arg^ [21,22] and then CAN could be misincorporated into proteins. CAN-containing proteins could lose their activity due to altered conformation, which can disrupt cellular metabolism [20].

In our previous research, we have indicated that CAN could be used as a convenient biochemical tool for the modification of NO metabolism in plant cells [20,23,24,25]. Although no typical mammalian NOS-like sequence was found in plant genomes [26], Arg-dependent production of NO in plants was demonstrated in many plant tissues [27]. CAN was clearly confirmed to inhibit Arg-dependent NO production (e.g., in apple (*Malus domestica* Borkh.) embryos [23] or in tomato roots [24]). CAN-induced decreased level of NO in tomato roots was accompanied by an oxidative burst manifested by increased superoxide radical production (O_2_^•−^) and hydrogen peroxide (H_2_O_2_) content as well as protein carbonyl groups accumulation [28,29]. This effect was exacerbated by the inhibition of key enzymes of the antioxidant cellular system (CAT, SOD, GR) [24]. On the contrary, during CAN-induced oxidative stress, no tremendous oxidative damage of membrane or DNA was observed [29]. Results of our research indicated that CAN increased total antioxidant capacity in tomato roots determined in methanol extract [24]. Therefore, we suspect that PCs could be the most important element contributing to total antioxidant capacity of tomato roots subjected to CAN application. In this context, the aim of our work was to determine how CAN-induced limitation of NO production and secondary oxidative stress impact PC content and metabolism in tomato roots. In this study, we have measured the total amount of PCs and analyzed histochemical localization of PCs and flavonoids in roots of tomato seedlings supplemented with this NPAA. Activities and gene transcription of two key enzymes of PC metabolism (PAL and PPO) were also investigated.

Overall, the results of this study indicated that CAN altered PC metabolism in tomato roots after long exposure to the NPAA, and PCs could act as main components of the cellular antioxidant system regulating ROS level, since they were accumulated in CAN-supplemented plants.

## 2. Results

### 2.1. Concentration of Phenolic Compounds in Tomato Roots Was Increased by Application of 50 µM CAN

**Figure 1:**
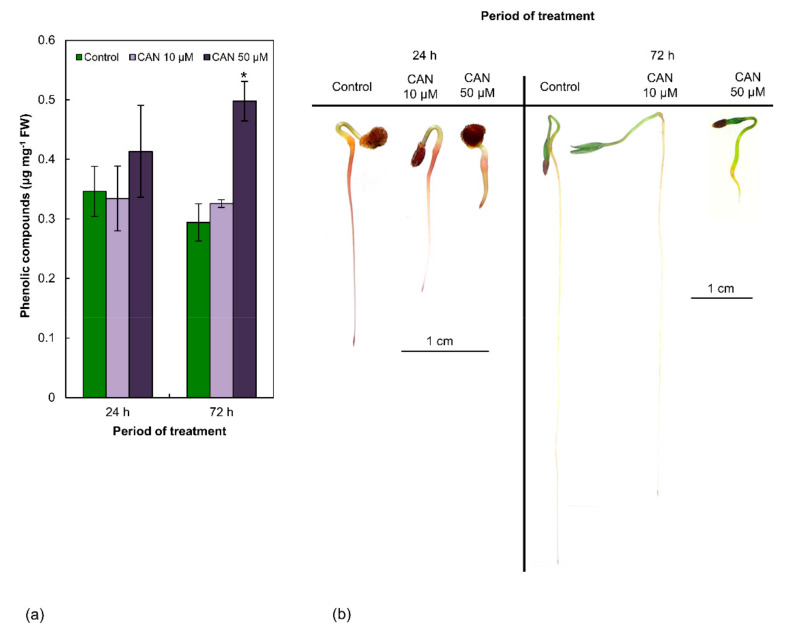
(**a**) The content of total PCs in roots of tomato plants grown in water (control) or supplemented with 10 or 50 µM CAN after 24 and 72 h of culture. Data were obtained in four independent experiments with three repetitions each. Asterisks (*) indicate significance between treatments and the control at the same time of culture period at *p* ≤ 0.05, based on Student’s test. (**b**) Localization of PCs (by Reeve’s method) in roots of tomato plants grown in water (control) or supplemented with 10 or 50 µM CAN after 24 and 72 h of culture. Data were obtained in three independent experiments, 10 roots from each treatment were used for localization of PCs.

Concentration of total PCs in control tomato plants was about 0.30–0.35 µg mg^−1^ FW (fresh weight) and was constant during the experiment (Figure 1a). CAN at lower concentration (10 µM) did not modify concentration of PCs in tomato roots, irrespectively of the duration of the treatment. After feeding with 50 µM CAN, concentration of total phenolics in roots increased in comparison to the control by 17% after 24 h and 70% after 72 h.

### 2.2. CAN at Higher Concentration (50 µM) Alters Localization of Phenolic Compounds in Roots of Tomato Seedlings

Staining of the roots of control tomato seedlings using Reeve’s method indicated the accumulation of PCs mostly in the root tips. The elongation parts of the roots were not stained, while the differentiation zone and roots primordia were visibly marked (Figure 1b). This pattern of phenolics visualization was typical in roots of plants grown in water or supplemented with CAN at low (10 µM) concentration, irrespectively of the duration of the culture. In contrast, treatment with 50 µM CAN resulted in uniform accumulation of phenolics all over the roots (Figure 1b). The more short-term (24 h) exposure of tomato seedlings to 50 µM CAN increased dark colorization of the roots’ apex (Figure 1b), which was not typical after prolongation of the treatment with CAN for an additional 48 h (Figure 1b).

### 2.3. CAN Increased Flavonoid Accumulation in Tomato Root Tips and Altered Flavonoid Localization in Root Tips

**Figure 2:**
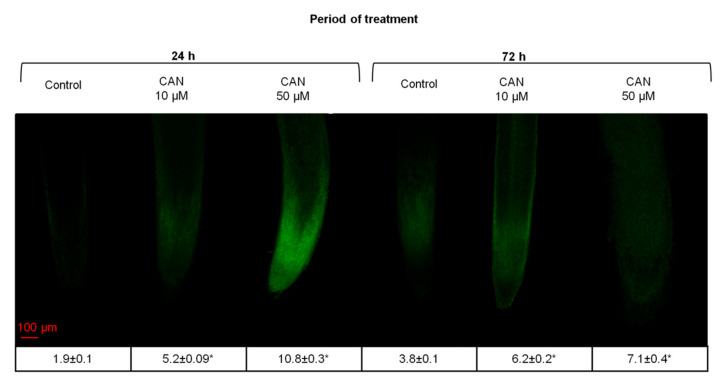
Localization of flavonoids with DPBA in roots of tomato plants grown in water (control) or supplemented with 10 or 50 µM CAN after 24 and 72 h of culture. Fluorescence intensity is shown in the table. Asterisks (*) indicate significance between treatments and the control at the same time of culture period at *p* ≤ 0.05, based on Student’s test. Data were obtained in three independent experiments, 10 roots from each treatment were used for flavonoid localization.

Flavonoids emit a very weak fluorescence with UV excitation but they can be distinguished from other phenols by treatment with 2-aminoethyl diphenylborinate (DPBA), also known as Naturstoffreagenz A. DPBA staining results in enhancement of the flavonoid autofluorescence. DPBA fluorescence is yellow when it originates from quercetin (Q-DPBA) or green when it refers to kaempferol (K-DPBA) [30,31]. In the root tips of all examined seedlings (both grown in water and supplemented with CAN), strong fluorescence signals of K-DPBA were observed, while Q-DPBA signal was brighter and less visible (Figure 2). No green or yellow fluorescence was observed in the negative control (root tips not treated with DPBA) (data not shown). In general, in roots of plants treated with CAN, the fluorescence signal of DPBA was stronger than in roots of plants grown in water (control), and increased in a dose-dependent manner (Figure 2). In roots of seedlings supplemented with 10 µM CAN for 24 or 72 h, the strong fluorescence was detected only in the elongation zone whereas the rest of the root exhibited the weak signal. In plants treated with 50 µM CAN for 24 h, the greater intensity of DPBA fluorescence occurred in the root cap, meristematic zone, and elongation zone. Root tips of seedlings fed with 50 µM CAN had the higher levels of flavonoid accumulation as judged by brighter fluorescence at equal exposure times. In tomato plants grown for 72 h with 50 µM CAN, flavonoids were localized along the root with clearly weaker signal in the elongation zone (Figure 2).

### 2.4. Activity of PAL in Tomato Roots Was Modified Only by 50 µM CAN

**Figure 3:**
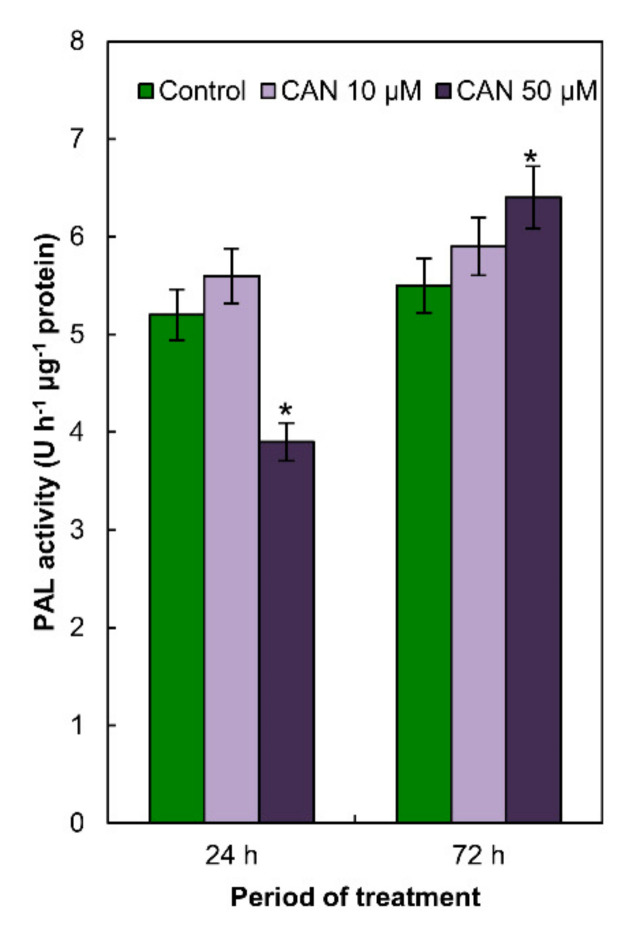
Activity of PAL in roots of tomato plants grown in water (control) or supplemented with 10 or 50 μM CAN after 24 and 72 h of culture. Data were obtained in three independent experiments with three repetitions each. Asterisks (*) indicate significance between treatments and the control at the same time of culture period at *p* ≤ 0.05, based on Student’s test.

PAL activity in the roots of control plants and roots of plants exposed to 10 µM CAN did not differ and was constant during the whole period of the experiment (Figure 3). CAN at higher (50 µM) concentration decreased (in 20%) PAL activity after short-term (24 h) treatment, while prolongation of the CAN application increased (in 20%) PAL activity in tomato roots (Figure 3).

### 2.5. Activity of PPO in Tomato Roots Increased Only after Prolonged Feeding with 50 µM CAN

**Figure 4:**
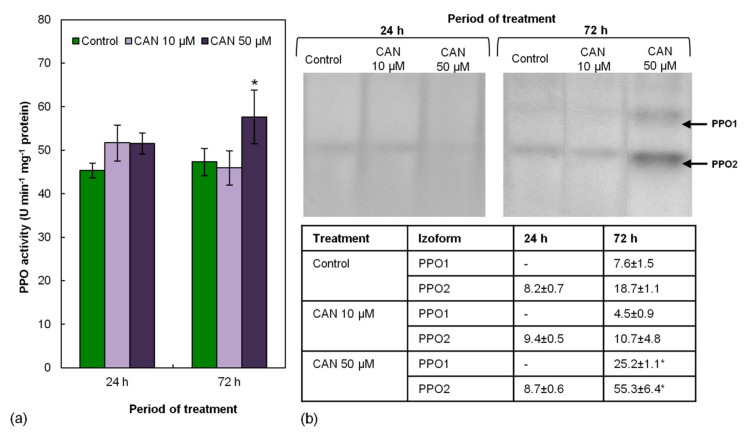
Activity of PPO in roots of tomato plants grown in water (control) or supplemented with 10 or 50 μM CAN after 24 and 72 h of culture, measured in (**a**) extract and (**b**) gel. Results of densitometric analysis are shown in the table. Data were obtained in three independent experiments with three repetitions each. Asterisks (*) indicate significance between treatments and the control at the same time of culture period at *p* ≤ 0.05, based on Student’s test.

Activity of PPO measured by a spectrophotometric method in root extracts of control plants was around 45 U min^−1^ mg^−1^ protein and remained constant during the experiment (Figure 4a). Short-term (24 h) treatment of tomato seedlings with CAN did not influence activity of PPO measured by the spectrophotometric method. Prolongation of the feeding of the plants with CAN increased PPO activity but only in extracts of the roots exposed to 50 µM CAN (Figure 4a).

Activity of PPO measured in the gel is shown in Figure 4b. No differences in PPO activity were observed after short-term application of CAN. At that time of the culture period, only one isoform (PPO2) was visible. After an additional 48 h of the culture in both control and CAN-treated roots, activities of two PPO isoforms (PPO1 and PPO2) were detected, but only 50 µM CAN significantly increased PPO1 and PPO2 activity.

### 2.6. CAN Modified Expression of Genes Coding PAL and PPO

**Figure 5:**
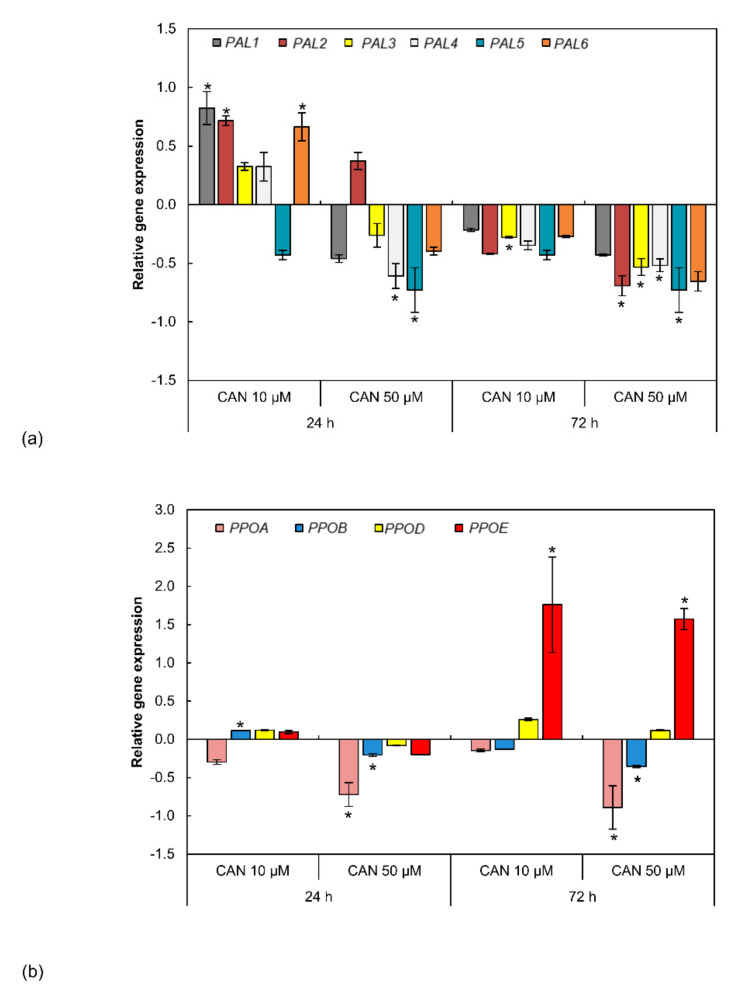
Expression level of genes encoding (**a**) PAL and (**b**) PPO in roots of tomato plants grown in water (control) or supplemented with 10 or 50 µM CAN after 24 and 72 h of culture. Data were obtained in four independent experiments with six repetitions each. Asterisks (*) indicate significance between treatments and the control at the same time of culture period at *p* ≤ 0.05, based on Student’s test.

PAL transcript levels in roots were determined for six genes (*PAL1-6*) (Figure 5a). Short-term supplementation with 10 µM CAN upregulated expression level of *PAL1*, *PAL2*, and *PAL6*, while transcription of other genes was not affected. Exposure of roots to 50 µM CAN for 24 h resulted in downregulation of *PAL4* and *PAL5*; transcript levels of four other tested genes were not changed. Prolongation of seedlings feeding with CAN led to downregulation of *PAL3* (irrespectively of CAN concentration), and downregulation of *PAL2*, *PAL4*, *PAL5* after 50 µM CAN; expression of other genes coding PAL was not modified (Figure 5a).

*PPO* transcript levels in roots were determined for four genes (Figure 5b). After 24 h of plants’ supplementation with 10 µM CAN, *PPOB* was upregulated, while expression of *PPOA*, *PPOD*, and *PPOE* was not changed. At the same time after supplementation with 50 µM CAN, *PPOA* and *PPOB* were downregulated, and expression of *PPOD* and *PPOE* was not modified (Figure 5b). Prolongation of roots’ exposure to 10 µM CAN resulted in a huge increase in the transcript level of *PPOE*, while expression of other genes was not changed (Figure 5b). Treatment of tomato with 50 µM CAN for 72 h downregulated *PPOA* and *PPOB*, upregulated *PPOE*, and had no impact on *PPOD* expression level.

## 3. Discussion

CAN belongs to the group of NPAA, whose action in plant cells is very wide because this group of chemicals is extremely heterogeneous. However, there are some data that show that NPAAs act in the plant defense reaction as key mediators or effectors in response to abiotic stresses [32] or biotic stresses due to their ability to modify ROS or reactive nitrogen species (RNS) production. Although induction of oxidative stress exhibited by overaccumulation of ROS and RNS in plants exposed to various NPAAs was demonstrated [24,25,33,34], the involvement of PCs in ROS scavenging or production was not investigated. Supplementation of tomato plants with CAN (10 or 50 µM) resulted in the restriction of elongation growth of the roots, as was previously described [24,29]. A similar response was observed also after various plants’ treatment with *meta*-tyrosine, another NPAA [33,35].

In general, tomato plants are considered a rich source of PCs and antioxidants. It applies not only to tomato fruits but also to other organs (stem, leaves, and roots) [36]. According to the literature, the concentration of PCs in tomato roots is relatively lowest in comparison to other parts of the plant. Flavonoids constitute about 50% of the total PCs in tomato roots, similar to leaves or stems [36]. In tomato roots exposed to CAN, elevated level of total PCs was observed only after prolonged culture in NPAA at higher concentration. It may suggest that the level of PCs in roots is sufficient to control elevated production of ROS occurring in tissue treated with 10 µM CAN or for a shorter period with 50 µM CAN. Silva-Beltran et al. [36] indicated a close correlation of antioxidant capacity of the tomato extracts to content of PCs and flavonoids. In our experiment, a similar pattern of an increased level of PCs and flavonoids to total antioxidant capacity determined by reduction of 2,2-diphenyl-1-picryhydrazyl (DPPH) [24] was observed. As indicated by several authors [36], tomato plants are rich in flavonoids and hydroxycinnamic acids. Among flavonoids identified in tomato, quercetin-3-rutinoside (rutin) is suggested to be the most predominant [36], and this compound is very effective in free radical scavenging. Localization of flavonoids with DPBA staining in tomato roots subjected to CAN pointed at the presence of quercetin and a huge enrichment of kaempferol, indicated as characteristic green fluorescence. Kaempferol, similarly to rutin, is well known for its prominent antioxidative activity [37]. In general, flavonoids can directly scavenge O_2_^•−^ and peroxynitrite (ONOO^−^) in an effective way, and they are known to block the reaction of free radicals in the NO signaling pathway in different cells and delay injury caused by free radicals [38]. Thus, taking into account that O_2_^•−^ and ONOO^−^ are suggested to be the key reactive species in CAN-supplemented tissue [25], the increased content of flavonoids was expected. Accumulation of total PCs or flavonoids accompanied by the increased antioxidant tissue activity seems to be the common response of plants to many environmental stress factors (e.g., drought or salinity [39,40,41,42,43]), although it has been rarely investigated in the case of allelopathy stress [44] or even never analyzed regarding toxicity induced by NPAAs of plant origin. Water extract of peppermint (*Mentha × piperita* L. CV. Mitcham) decreased content of total PCs in tomato seedlings [44]. On the other hand, there are a lot of data referring to the allelopathic potential of different PCs, pointing at an important role of PCs, particularly flavonoids, in root morphology, mainly due to modification of polar auxin transport [45,46,47]. In sorgo (*Sorghum bicolor* (L.) Moench) roots’ inhibition of growth, a higher number of lateral roots after treatment with rutin or IAA, was observed. The results were similar for the plants exposed to leaf extracts of diesel tree (*Copaifera langsdorffii* Desf.) containing as the major compounds quercetin-3-*O*-alpha-rhamnoside and kaempferol-3-*O*-alpha-rhamnoside [48]. Therefore, we cannot exclude that malformations in growth of roots of CAN-supplemented tomato seedlings (particularly exposed to 50 µM NPAA) result from increased level of flavonoids, especially since increased IAA content was previously noticed in roots of tomato seedlings fed with CAN [29].

PAL converts Phe to trans-cinnamic acid, which is the precursor for the synthesis of most PCs. The increased activity of PAL is correlated with an elevated level of PCs. In addition, PAL is considered as a marker of environmental stresses in many plants, as stimulation of the activity of the enzyme is typical for various stresses [49]. Some allelochemicals belonging to the group of PCs such as ferulic acid or *p*-coumaric acid have increased the activity of PAL [50]. Data presented by Omezzine et al. [51] indicated various effects of fenugreek (*Trigonella foenum-graecum* L.) extracts on activity of PAL in lettuce (*Lactuca sativa* L.) roots and leaves. In our experiment, application of CAN at lower concentration had no effect on PAL activity in tomato roots, but 50 µM CAN after 24 h treatment slightly inhibited PAL, whereas prolonged culture in 50 µM CAN stimulated activity of the enzyme. In general, it corresponds well to inhibition of tomato root growth, which was completely blocked after 72 h supplementation with 50 µM CAN. This observation is in agreement with other research, demonstrating that the increased activity of PAL after treatment with allelochemicals such as derivatives of cinnamic and benzoic acids was accompanied by inhibition of root growth in maize (*Zea mays* L.) [52], cucumber (*Cucumis sativus* L.) [53], or soybean (*Glycine max* (L.) Merr.) [54]. In addition, slightly higher activity of PAL in roots of tomato treated with 50 µM CAN corresponds to increased content of PCs and deposition of PCs in roots. Although it is commonly accepted that the overproduction of ROS correlates to enhancement of PAL activity, Kovacik et al. [55] have shown that in N-deficient plants, application of ROS scavengers increased activity of PAL. After testing NO scavenger (PTIO), they found decreased PAL activity and the content of the total soluble PCs, whereas exogenous application of H_2_O_2_ and NO stimulated PAL activity and accumulation of phenols. Similarly in tobacco bright-yellow2 cells, only application of sodium nitroprusside (SNP) as NO donor, together with ROS generator (Glc plus Glc oxidase), led to increased PAL activity [56]. Involvement of NO in regulation of PAL was investigated also in pearl millet (*Pennisetum glaucum* (L.) R. Br.) treated with elicitors isolated from *Pseudomonas fluorescens* [57]. Both enzymatic activity and expression of the genes encoding PAL were decreased after application of NO scavenger [57]. Stimulation of PAL activity by SNP and lowering of activity in the presence of NO scavenger were detected in wheat (*Triticum aestivum* L.) leaves [58]. Manjunatha et al. [59] demonstrated that NO effectiveness in protecting pearl millet (*Pennisetum glaucum* L.) R. Br.] plants against downy mildew disease caused by *Sclerospora graminicola* [(Sacc). Schroet] is linked to enhancement of PAL by NO donor. Although CAN slightly modified activity of PAL in roots of tomato plants, its impact on transcription level of the genes encoding the enzyme was more visible. Only 10 µM CAN supplemented for a short period upregulated almost all PAL genes (*PAL1, PAL2, PAL3, PAL4*, and *PAL6*), while CAN at higher concentration or prolongation of the NPAA treatment resulted in downregulation of the genes. Thus, we do not observe the same pattern of transcript level and enzymatic activity. CAN inhibited Arg-dependent NOS-like activity [24] and lowered NO emission in tomato roots [28], therefore decreased expression level of genes encoding PAL is in agreement with the previous data, reporting that inhibitors of NO synthesis or NO scavengers downregulate *PAL* expression (e.g., in Arabidopsis leaves infected by *Pseudomonas syringae* pv. *glycinea* [60]). It is suggested that a simultaneous ROS and NO burst determines upregulation of genes encoding PAL [61,62]. Thus, it is possible that elevated production of ROS [28] observed in roots after CAN application is not sufficient for enhancement of *PAL* expression because of the shortage of NO.

The sorghum extract (sorghaab) exerted the same effect on activity of PAL and PPO in sesame (*Sesamum indicum* L.), black-jack (*Bidens pilosa* L.), and goose grass (*Eleusine indica* L. Gaertn); stimulation of PAL was accompanied by stimulation of PPO activity [63]. The pattern of changes in enzymatic activity of PPO and PAL in tomato roots exposed to CAN also was identical after 72 h of the culture—about 20% increase of activity was noticed in 50 µM CAN, whereas PPO activity was not modified by CAN after 24 h. Stimulation of PPO activity seems to be a typical response of plants to biotic stress, particularly wounding and pathogens, and determines resistance to stress. In a very elegant way it was described by Thipyapong et al. [64] on the background of transgenic tomato plants with overexpression or suppression of PPOs. Stimulation of PPO activity was also observed in tomato during drought stress [65]. Although PPO is a common enzyme in plants and its role in browning of the tissue due to the oxidation of *o*-phenols to *o*-quinones is well known, PPO’s function in plant development is still unclear [13]. It seems to be activated by typical stress hormones or regulators such as jasmonic acid (JA), ethylene, ABA, salicylic acid, or systemin [64]. Its activation can vary depending on the plant species (e.g., in two cultivars of wheat, ABA did not modify PPO or lead to inhibition of the activity [66]). In addition, the influence of NO on PPO is uncertain. In our experiment, lowering production of NO in tomato roots due to limitation in Arg-dependent NO synthesis had no or only slight effect on stimulation of PPO activity. In roots of wheat, application of NO scavenger increased activity of PPO only in the cell wall fraction [66]. It was suggested that PPO may act in cell wall stiffening. In contrast, SNP decreased PPO activity in roots of wheat, while in shoots, the effect of this NO donor was diverse. Therefore, based on lack of or vague impact of NO on PPO activity, we can conclude that NO is probably not a regulator of PPO, although in plants response to pathogens, plays an important signaling role [67,68].

Despite NO limitation, application of CAN may be considered also as a biotic stress, because this NPAA can act as an allelochemical or phytotoxin. In virus-infected sunflower (*Helianthus annuus* L.) leaves, the increased activity of PPO was accompanied by increased POx activity [69]. CAN supplementation stimulated POx activity [28], thus the same pattern of modification in POx and PPO activities was observed in tomato roots. The correlation between PPO and POx activities was demonstrated in leaves and steams of common jasmine (*Jasminum officinale* L.) after infection with *Uromyces hobsoni* [70]. POx and PPO were also involved in resistance to bacterial blight in rice (*Oryza sativa* L.) varieties; upon inoculation with *Xanthomonas oryzae* pv. *oryzae* in which activities of both enzymes were elevated [71]. It was concluded that activities of both phenol-oxidizing enzymes, PPO and POx, in the infected tissues were necessary for plant response to fungus or bacteria, and enhanced oxidation of polyphenols may act as a biochemical barrier for the development of the pathogen. This conclusion, of course, cannot be the explanation of the role of PPO in the context of CAN toxicity, but its increased activity leading to oxidation of polyphenols could lead to modification of cell wall structure resulting finally in inhibition of cell elongation and restriction in root growth [29]. In our experiment, after 24 h of the culture of tomato plants, activity of only one isoform of PPO (PPO2) was observed in control and CAN-supplemented seedlings. Prolongation of the experiment resulted in appearance of the activity of the second PPO isoform (PPO1). It is possible that PPO1 was present in the latent form in roots of younger seedlings and was activated during development. Moreover, CAN, similar to other stress factors [72], stimulated the activity of both isoforms after 72 h treatment.

In tomato roots, we measured also the expression level of four genes encoding PPOs: *PPOA*, *PPOB*, *PPOD*, and *PPOE*. *PPOC* and *PPOF* were not analyzed because they are not expressed in tomato roots [12]. Upregulation of *PPOE* was noticed after prolonged CAN supplementation, while downregulation of *PPOA* was detected just after a short period of the treatment. According to the literature, the highest *PPO* expression levels are associated with young tissues and meristematic regions, and the gene expression generally declines during development and maturation of the plant tissues [64]. In some plants, PPOs are very stable during the ontogeny and often exist in a latent form in tissues even when *PPOs* transcripts are no longer found [64]. Therefore, we were not surprised that the pattern of *PPO* gene expression and PPO activity were not identical. PPOA exhibits hydrophobic characteristics and is therefore able to interact with membranes, whereas PPOE is proposed to be more soluble [73]. Kampatsikas et al. [73], investigated biochemical and structural features of these two PPOs tomato isoforms. They concluded that PPOA has tyrosinase activity (TYR) while PPOE is catechol oxidase (CO). TYRs catalyze the *o*-hydroxylation of monophenols (monophenolase activity, EC 1.14.18.1) and the subsequent two electron oxidation of the resulting *o*-diphenols to the corresponding *o*-quinones (diphenolase activity, EC 1.10.3.1), whereas COs are only capable of catalyzing the latter diphenolase reaction [73].

In potato (*Solanum tuberosum* L.) tubers it was shown that individual or cumulative downregulation of one *PPO* gene did not usually cause upregulation of the other *PPO* genes [74]. Authors suggested that different *PPO* genes may be regulated independently reflecting their diversified functions. It was also shown in Chinese sage (*Salvia miltiorrhiza* Bunge) that the expression of *PPOs* was responsive to MeJA treatment and other stress factors [75]. Similarly, many different stress factors (H_2_O_2_, low temperature, salinity, and other biotic stressors) upregulated expression of two genes encoding PPOs (*FaPPO1* and *FaPPO2*) in strawberry (*Fragaria × ananassa* Duch.) fruits [76]. Therefore, it is possible that genes encoding proteins of COs activity may be preferentially upregulated under CAN stress, and thus can lead to enhanced formation of lignin and quinone.

Investigation of the PCs content and PCs oxidation in roots of plants with lowered NO level, due to inhibition of NO biosynthesis, has never been conducted before, although it is of great interest. In mammalian tissues, PCs originated from plants act as inhibitors of nitric oxide synthase (NOS) [77] It is clear that Arg-dependent biosynthesis of NO in plants has no similarities to the NOS path in mammalian cells, thus the relationship of PCs with NO metabolism in plants could be different. We believe CAN to be a phytotoxic compound inducing overproduction of ROS in plants, therefore we suggest that elevated level of PCs could act as sufficient antioxidants preventing formation of cellular deterioration due to oxidative stress. Increased oxidation of PCs due to higher activity of PAL and PPO could lead to malformation in cell wall structure and result in a restriction in root growth under CAN supplementation.

## 4. Materials and Methods

### 4.1. Plant Material

Tomato seeds (*Solanum lycopersicum* L. cv. Malinowy Ożarowski), obtained from PNOS Sp. z o.o., were germinated on Petri dishes filled with moistened filter paper at 20 °C in darkness for three days. Then, seedlings with roots 5 mm long were selected and transferred to Petri dishes (Ø15 cm), which were filled with filter paper wetted with distilled water (control) or CAN (L-stereoisomer, Sigma-Aldrich) dissolved in distilled water. CAN at concentrations of 10 and 50 µM was applied for 24 or 72 h according to [29].

### 4.2. Total Phenolic Compounds (PCs) Measurement

The level of total PCs in tomato roots was measured using Folin-Ciocalteu reagent. Roots of tomato seedlings (0.1 g) were homogenized with mortar and pestle in 0.5 mL 80% (*v/v*) methanol. PCs were extracted in ultrasonic bath for 5 min at 4 °C. Extracts were centrifuged at 7000× *g* for 10 min at 4 °C. To 100 μL of supernatant was added 700 μL of diluted (1:10 in distilled water) Folin-Ciocalteu reagent (Sigma-Aldrich). The mixture was incubated for 5 min in darkness at room temperature. After incubation, 100 μL of 7.5% (*w/v*) sodium carbonate solution was added and the mixture was incubated for another 30 min in the same conditions. Content of total PCs was measured at λ = 765 nm with spectrophotometer Hitachi U-2900. The standard curve was prepared using gallic acid (Sigma-Aldrich). Content of total PCs was expressed as mg g^−1^ FW. Experiments were done in three independent replicates with three repetitions in each.

### 4.3. Histochemical Localization of PCs

The localization of PCs was done according to Reeve [78]. Equal volumes of 10% (*w/v*) sodium nitrite, 20% (*w/v*) urea, 10% (*v/v*) acetic acid were mixed and tomato seedlings were immersed in the mixture for 4 min in darkness. Yellow coloration and gas evolution appeared immediately. Next, two volumes of 2 M NaOH were added and the color was changed to dark brown. Seedlings were briefly dried on a paper towel and images were taken with TAGARNO FHD TREND digital microscope with magnification 5.5×, aperture f/5.6, shutter speed 1/90, enhancement 20.5. PCs react with nitrous acid and color products are formed. Control was obtained by reversing the acid and base in the procedure. Data were obtained in three independent experiments, 10 roots from each treatment were used for PCs localization. The representative images were shown.

### 4.4. Flavonoids Localization

Flavonoids localization was performed as described by [79] with 2-aminoethyl diphenylborinate (DBPA). Roots were isolated and immediately submerged in an aqueous 0.01 mM DBPA solution with 0.01 (*v/v*) Triton x-100 for 7 min. Then, roots were washed with distilled water. Roots were mounted in distilled water and imaged on a confocal laser scanning microscope Leica TCS SPSII (Leica Microsysem CMS, Wetzlar, Germany) with excitation at 458 nm and emission 650 nm for kaempferol and 515 nm for quercetin. Data were obtained in three independent experiments, 10 roots from each treatment were used for flavonoids localization. Representative images were shown.

### 4.5. Measurement of PAL Activity

PAL activity was determined according to [80]. Roots of tomato seedlings (50 mg) were homogenized with mortar and pestle in 1 mL 0.1 mM Tris-HCl pH 8.5 with 5 mM DTT, 1 mM EDTA, 10 mM PMSF, and 5% (*v/v*) glycerol in an ice bath. Homogenates were centrifuged at 10,000× *g* for 10 min at 4 °C. The supernatant was collected and used in further analysis. Reaction mixture containing 1 mL 100 mM Tris-HCl pH 8.5, 0.5 mL 1 mM Phe, and 0.5 mL enzymatic extract was incubated at 40 °C for 1 h. The reaction was stopped by adding 50 μL 5 M HCl. Absorbance was measured at λ = 290 nm with spectrophotometer Hitachi U-2900. Results were expressed as U h^−1^ μg^−1^ protein.

### 4.6. Preparation of the Protein Extract for PPO Activity Measurement

Roots of tomato seedlings (200 mg) were homogenized with mortar and pestle in 0.5 mL 0.2 M K-phosphate buffer pH 7.0 with 0.2 M NaCl, 0.25% Triton x-100, 1% (*v/v*) protease inhibitor cocktail (Sigma-Aldrich P9599) and 5% (*w/v*) PVPP in an ice bath. After centrifugation at 10,000× *g* for 10 min at 4 °C, supernatants were desalted with protein concentrator PES, 3 K MWCO (Thermo Scientific^TM^) at 10,000× *g* for 30 min at 4 °C. After desalting, supernatants were collected for further analysis.

### 4.7. Measurement of PPO Activity

#### 4.7.1. Measurement of PPO Activity in the Root Protein Extract

PPO activity was measured according to [81]. The reaction mixture containing 258 μL 50 mM K-phosphate buffer pH 7.5 and 3 μL of protein extract was preincubated for 5 min at 30 °C. Next, 30 μL of 50 mM 4-tetr-butylcatechol (BC) and 9 μL 25 mM 4-amino-N,N-diethylaniline (ADA) was added. The total volume of the assay mixture was 300 μL. Absorbance at λ = 625 nm was measured for 3 min. PPO activity was expressed as U min^−1^ mg^−1^ protein.

#### 4.7.2. Measurement of PPO Activity in the Polyacrylamide Gel under Nondenaturing Conditions

PPO activity was analyzed in a gel according to [82]. Soluble proteins (10 μg with 60% glycerol in ratio 3:1) samples were separated using 8% polyacrylamide gel electrophoresis under nondenaturing and nonreducing conditions at 4 °C. After electrophoresis, gels were soaked in 0.1 M K-phosphate buffer pH 7.0 for 3 min at RT, followed by incubation in 20 mM acetic acid with 25 mM BC at RT until yellow bands appeared. Immediately gels were rinsed with 0.1 M K-phosphate buffer pH 7.0. Next, gels were incubated in 10 mM HCl with 25 mM ADA until bands became dark blue. Images of representative gel were shown. Original image of the gels are shown in Figure S1.

### 4.8. Protein Content Quantification

Protein level in tissue extracts was determined by the Bradford method [83] using bovine serum albumin as a standard.

### 4.9. PPO and PAL Gene Expression Analysis

The gene expression was determined in roots using a quantitative real-time polymerase chain reaction (qRT-PCR). Total RNA was extracted and purified with an RNAzol RT (Sigma-Aldrich) according to producer’s instructions/manufacturer’s manual. RNA samples were treated with DNase I (Thermo Scientfic^TM^). The amount of 200 ng of total RNA was used to generate cDNA by ReverteAid First Strand cDNA Synthesis Kit (Thermo Scientific^TM^) with oligo(dT)18 Primer in a 35 μL total volume, as is described in the manufacturer’s procedure. qRT-PCR was performed in a CFX Connect^TM^ Real-Time PCR System. iTaq^TM^ Universal SYBR^®^ Green Supermix (Bio-Rad) was applied as the basis for the reaction in a total volume of 12 μL (6 μL PCR Supermix, 1 μL primer, 4 μL H_2_O, and 1 μL cDNA). Table S1 shows the primer pairs used to amplify the genes of PPO and PAL. For the normalization of the expression levels, two housekeeping genes, APN and PP2A (stability value for the best combination of two genes was 0.006 determined by NormFinder software, five potential reference genes were tested), were used as reference genes; cDNA from untreated material was used as a reference sample.

### 4.10. Densitometry Analysis

Densitometry analysis and analysis of fluorescence intensity were done using Image J.

### 4.11. Statistics

Data were obtained in at least three independent experiments with at least three repetitions each. All data were analyzed using Statistica Software. Mean differences were calculated using *t*-test; SD was also provided to indicate the variations associated with the particular mean values.

## Supplementary Materials

The following are available online at https://www.mdpi.com/2223-7747/9/11/1595/s1, Figure S1: Original image for the gel Figure 4b in the manuscript, Table S1: List of primers used for RT-qPCR experiments.
