# Supplementary material for: Canavanine Increases the Content of Phenolic Compounds in Tomato (Solanum lycopersicum L.) Roots
Source: Plants (Basel). 2020 Nov 17;9(11):1595. doi: 10.3390/plants9111595 (PMC7698470; doi:10.3390/plants9111595)
Supplement: Supplementary file 1 [file plants-09-01595-s001.pdf]

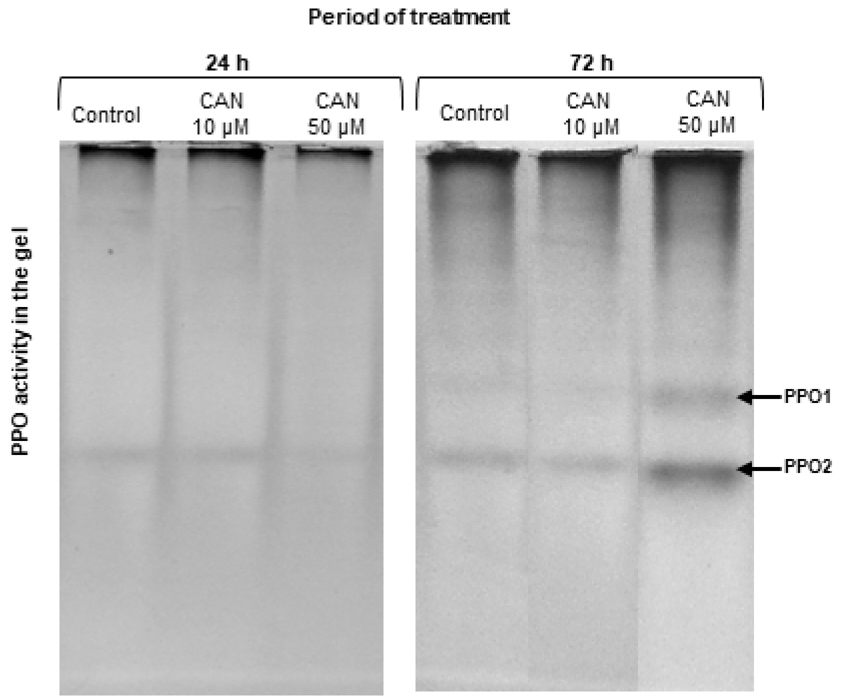

Fig S1. Original image for the gel Fig.4.b in the manuscript.

Table S1. List of the primers used for RT-qPCR experiments

Gene name	Gene ID (NCBI)	Primers sequences 5'-3'	PCR product length (bp)
PAL1	XM_026029821.1	F: ACTTGTGAATGGCACAGCAG<br>R: GGCTTTCCGTTCATCACTTC	123
PAL2	NM_001320601.1	F: GTTTGCCTTCGAATCTCACC<br>R: TACGAGGCCATAGCGATTTC	82
PAL3	NM_001320609.1	F: GAGATCGACAAGGTGTTCACAG<br>R: TTTCTGGCAAGCATCTAGCA	116
PAL4	XM_004246601.3	F: CGGTGAGGAGATTGACAAGG<br>R: CCATTCCAGCTCTTGAGACAC	87
PAL5	NM_001320040.1	F: CTGGCAGGCCTAATTCAAAG<br>R: AACACCAGCCACATGAAACG	80
PAL6	XM_004249510.4	F: TGAGACGTTGAATGCTGAGG<br>R: AGCCAAACCAGAACCAACAG	121
PPOA	XM_004245981.4	F: ATTTGGGTTGGTACGAGACG<br>R: ATTCGTTCCACATCCTGTCC	142
PPOB	NM_001309397.1	F: GCTATCAACAGGCCAGCTTC<br>R: ATCATTCTCGCCAGCTCTGT	219
PPOD	NM_001309397.1	F: TTGGCGGACCTTATGATCTC<br>R: TGGGCAAAGTTGAACCTCTC	114
PPOE	XM_004245993.4	F: CACCACTCCCAAAAGACCTAAC<br>R: GTGCGGCTGGTCTAATTTTG	81
